# Prefrontal cortex modulates the correlations between brain-derived neurotrophic factor level, serotonin, and the autonomic nervous system

**DOI:** 10.1038/s41598-018-20923-y

**Published:** 2018-02-07

**Authors:** Wei Hung Chang, I Hui Lee, Mei Hung Chi, Shih-Hsien Lin, Kao Chin Chen, Po See Chen, Nan Tsing Chiu, Wei Jen Yao, Yen Kuang Yang

**Affiliations:** 1 Department of Psychiatry, National Cheng Kung University Hospital, College of Medicine, National Cheng Kung University, Tainan, Taiwan; 2 Institute of Clinical Medicine, College of Medicine, National Cheng Kung University, Tainan, Taiwan; 3 Department of Nuclear Medicine, National Cheng Kung University Hospital, College of Medicine, National Cheng Kung University, Tainan, Taiwan; 4 Department of Psychiatry, National Cheng Kung University Hospital, Dou-Liou Branch, Yunlin, Taiwan; 5 Institute of Behavioral Medicine, College of Medicine, National Cheng Kung University, Tainan, Taiwan

## Abstract

Top-down regulation in the human brain and anatomical connections between the prefrontal cortex (PFC) and specific catecholamine-related regions have been well-studied. However, the way in which the PFC modulates downstream neuro-networks in terms of serotonin and the autonomic nervous system (ANS) by variation in the level of brain-derived neurotrophic factor (BDNF) is still unclear. We recruited sixty-seven healthy subjects. Serotonin transporter (SERT) availability was examined by SPECT with [^123^I]ADAM analysis; heart rate variability (HRV) testing was performed, and the BDNF level was measured. The Wisconsin card-sorting test (WCST), which assesses PFC activation, was also conducted. The interactions of BDNF level and SERT availability were significant in relation to the HRV indexes of low frequency, high frequency, total power, and mean heart rate range. Moderate to significant positive correlations between SERT availability and the above-mentioned HRV indexes existed only in subjects with a low BDNF level. Furthermore, in the low BDNF level group, only those with high WCST perseveration errors or low category completions exhibited significant positive correlations between SERT availability and HRV indexes. A lower BDNF level and poorer PFC function might modulate the synergistic effects of serotonergic and ANS systems in order to maintain brain physiological and psychological homeostasis.

## Introduction

The human prefrontal cortex (PFC) manages several aspects of cognitive- emotional-behavioral regulation and plays crucial roles in problem-solving, strategy-shifting and error-monitoring^1^. In addition, top-down regulation from the PFC is connected to different brain regions and exerts neuromodulatory effects under stressful environments, which involve evocation of several neurochemical factors to orchestrate brain activities^2^.

For example, a high level of brain-derived neurotrophic factor (BDNF) in the PFC has been found to prevent or reverse stress-related mental illness by enhancing stress resilience^3^. Furthermore, BDNF can cross the blood-brain barrier, and its levels in serum and plasma are highly correlated with its level in the cerebrospinal fluid^4^. Serotonin is an important monoamine that manages several important domains of human cognition, emotional regulation, and behavior responses, and even exerts neuromodulatory effects under stress^5^. The serotonin transporter (SERT), which transports excessive serotonin from the synapse cleft to pre-synapse neurons, is important in terms of its antidepressant effects^6^ and several serotonin-related activities^7^, and altered SERT availability has been observed in major depressive disorder (MDD) and other anxiety disorders. More importantly, a connection between the PFC and the dorsal raphe nucleus (DRN), which is abundant in serotonin, has been demonstrated during stress management^8^. In addition, a previous study showed that SERT function is modulated by BDNF^9^, added to which the interaction effects of BDNF and serotonin have been demonstrated to be involved in the management of emotional regulation^10^.

The above-mentioned neurobiological systems that manage stress under challenging environments may be clinically measurable by the autonomic nervous system (ANS) performance, which alters human psycho-physiological conditions directly in order to cope with stress (fight or flight)^11^. For example, high frequency (HF) heart rate variability (HRV) predicts depressive symptoms in adolescents^12^, and reduction in HRV may lead to a greater risk of cardiovascular disease^13^. In addition, mentally perceived stress was found to be negatively correlated with the HF component of HRV^14^. Correlation between serotonin and the ANS, both of which have similar regulatory functions, has been reported previously^15–19^. Structurally, serotonergic neurons originating from the raphe nucleus in the midbrain project onto the nucleus tractus solitarius, which is responsible for cardiovascular reflexes and ANS function^15,16^. Clinically, the tryptophan depletion test, which decreases the serotonin level, may lead to lower HRV and more severe depressive symptoms^17^. Our previous study also showed that the effect of anxiety on HRV is related to serotonin vulnerability^20^. The antidepressant effect also alters HRV and may increase the risk of cardiovascular disease^18^. Genetically, serotonergic gene polymorphism modulates parasympathetic activity under conditions of greater stress^19^. Although there exists no direct evidence of a correlation between BDNF and the ANS, genetic analysis has shown that BDNF genes influence sympathovagal tone^21^. Moreover, the PFC modulates ANS function when facing physiological or psychological stress^22^.

To conclude, besides the ascertained structural connection, a frontal-subcortical functional connection and connections between subcortical activities might exist. However, the effects of BDNF and serotonin, as mentioned in previous studies, on the ANS remain unclear, and how regulatory effects of the PFC modulate downstream effects needs to be clarified. Therefore, we used [^123^I]-labeled 2-((2-((dimethylamino)methyl) phenyl)thio)-5- iodophenylamine ([^123^I]ADAM), a radioligand with a high selectivity and a strong affinity to SERT, combined with the techniques of single-photon emission computed tomography (SPECT), to examine SERT availability in the midbrain, and employed HRV frequent domain measurement to represent ANS function. We also used the Wisconsin card-sorting test (WCST), which is a psychological test that assesses prefrontal cognitive flexibility and executive function, and is correlated with stress management^23^. Hence, our study aimed to (i) explore the possible effects of BDNF and serotonin availability on the ANS; (ii) identify correlations among serotonin, each HRV component (subcortical-subcortical correlations), and BDNF level; and (iii) investigate the modulatory effect of the PFC in terms of WCST performance on the above associations (frontal-subcortical associations).

## Results

### Demographic data and interactions of BDNF level and SERT availability with HRV indexes

Table 1 presents the basic demographic data for the whole group of normal healthy participants. In line with the aims of the present study, we first examined the interactions of BDNF level and SERT availability with ANS function, and significant interactions were found with LF (*F* = 4.00, *p* = 0.0497), HF (*F* = 4.20, *p* = 0.045), total power (*F* = 4.53, *p* = 0.037), and MHRR (*F* = 6.42, *p* = 0.014) (Table 2). Full results of the interactions of BDNF level and SERT availability with ANS functions are presented in Supplemental Table 1. Subjects were divided into 2 groups according to the median BDNF level for further analysis. Differences in the demographic and other variables between the 2 groups are shown in Table 1. Besides the significant BDNF level difference due to stratification, only LF/HF was significantly higher in the low BDNF level group than in the high BDNF level group (*p* = 0.01).

**Table 1:** Demographic data and comparison of low and high BDNF level groups.

	Total (*n* = 67)		Low (*n* = 33)		High (*n* = 34)		Statistic	
	Mean	± SD	Mean	± SD	Mean	± SD	*t*/*χ*^2^	*p*
Age	36.10	±13.30	34.09	±12.85	38.06	±13.62	−1.23	0.22
Sex (M/F)	26/	41	14/	19	12/	22	0.36	0.55
Smoking (Y/N)	6/	61	3/	30	3/	31	0.00	0.97
Alcohol (Y/N)	5/	62	2/	31	3/	31	0.19	0.67
Educational years	13.31	±3.83	12.91	±4.64	13.71	±2.86	−0.85	0.40
Body mass index	22.92	±3.79	23.52	±4.41	22.33	±3.02	1.29	0.20
Systolic blood pressure (mmHg)	115.63	±16.02	116.10	±16.26	115.21	±16.05	0.22	0.83
Diastolic blood pressure (mmHg)	73.94	±10.47	75.27	±10.88	72.73	±10.09	0.96	0.34
ln (LF)	5.86	±0.82	5.89	±0.91	5.83	±0.75	0.31	0.76
ln (HF)	5.36	±1.06	5.18	±1.10	5.55	±1.00	−1.45	0.15
ln (LF/HF)	0.50	±0.65	0.72	±0.56	0.28	±0.67	2.85	0.01
ln (total power)	7.07	±0.78	7.04	±0.86	7.10	±0.70	−0.32	0.75
MHRR	13.70	±5.63	13.35	±5.47	14.05	±5.84	−0.51	0.61
SERT availability	2.18	±0.54	2.06	±0.48	2.30	±0.58	−1.80	0.08
BDNF (pg/ml)	9178	±5652	4613	±2294	13610	±4207	−10.82	<0.001

Note: subjects were split into low and high BDNF level groups according to the median (8,500 pg/ml).

BDNF: brain-derived neurotrophic factor.

PSQI: Pittsburgh sleep quality index.

RLCQ: recent life change questionnaire.

LF: low frequency.

HF: high frequency.

MHRR: mean heart rate range.

SERT: serotonin transporter.

**Table 2:** Interactions of BDNF level and SERT availability with HRV indexes and Spearman’s rho correlations between SERT availability and HRV indexes in subject groups with low and high BDNF levels.

	BDNFхSERT^a^		SERT			
			Low BDNF (*n* = 33)		High BDNF (*n* = 34)	
	*F*	*p*	*ρ* ^b^	*p*	*ρ* ^b^	*p*
ln (LF)	4.00	0.0497	0.49	0.004	0.07	0.68
ln (HF)	4.20	0.045	0.43	0.012	−0.04	0.80
ln (LF/HF)	0.65	0.42	−0.07	0.69	0.09	0.62
ln (total power)	4.53	0.037	0.47	0.006	0.04	0.82
MHRR	6.42	0.014	0.68	<0.001	0.01	0.94

^a^The results were similar if the BDNF group was used instead of BDNF level.

^b^The results were still significant if partial correlation was used after controlling for age and sex, except ln (HF) (*r* = 0.30, *p* = 0.10).

Note: subjects were split into low and high BDNF level groups according to the median (8,500 pg/ml).

BDNF: brain-derived neurotrophic factor.

SERT: serotonin transporter.

HRV: heart rate variability.

LF: low frequency.

HF: high frequency.

MHRR: mean heart rate range.

### Correlations between SERT availability and HRV indexes in subjects with different BDNF levels

We examined the correlations between SERT availability and HRV indexes in the different groups. The results showed that SERT was significantly correlated with LF (*ρ* = 0.49, *p* = 0.004), HF (*ρ* = 0.43, *p* = 0.012), total power (*ρ* = 0.47, *p* = 0.006), and MHRR (*ρ* = 0.68, *p* < 0.001) in the low BDNF group, but not in the high BDNF group. These significant correlations still existed after controlling for age and sex, with the exception of the marginal significant correlation with HF (*ρ* = 0.30, *p* = 0.10) (Table 2).

### WCST modulated the correlations between SERT availability and HRV indexes in different BDNF level groups

**Figure 1:**
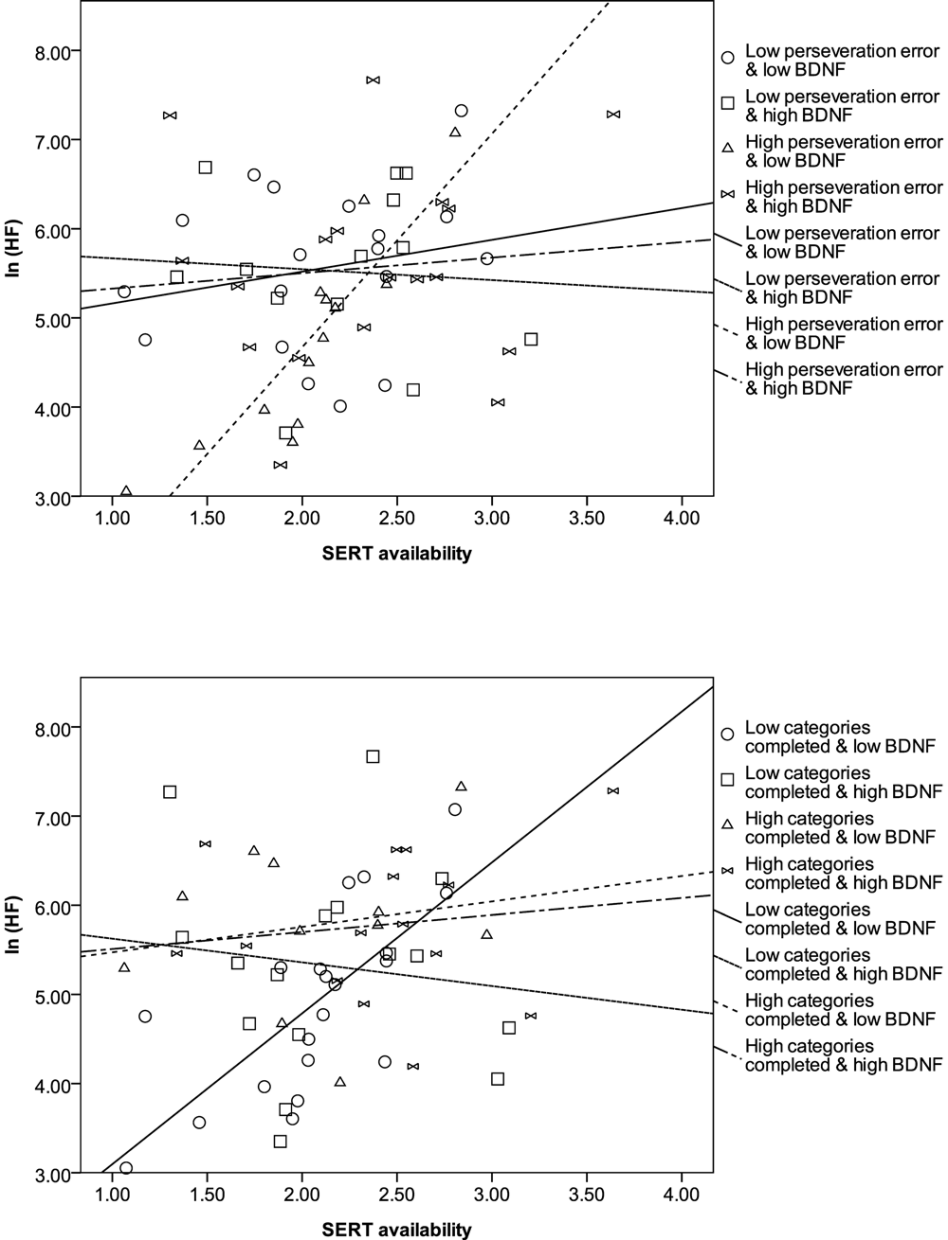
Correlation between SERT and ANS function in subjects with different BDNF levels, and modulation effects of PFC function in terms of WCST examination. Parasympathetic predominant domains may be positively correlated with SERT in subjects with a low BDNF level and poorer PFC function.

We examined whether these findings were similar in groups with different levels of prefrontal cognitive flexibility function, as assessed by the two performance indexes of the WCST. The results showed that only subjects with a low BDNF level and a poorer WCST performance (higher perseveration errors or lower categorical completion) exhibited significant positive correlations between SERT availability and HRV indexes (see Tables 3 and 4, and Fig. 1).

**Table 3:** Spearman’s correlations between SERT availability and HRV indexes in low and high BDNF level groups modulated by the perseveration error of WCST.

	SERT availability							
	Low perseveration error				High perseveration error			
	Low BDNF (*n* = 18)		High BDNF (*n* = 16)		Low BDNF (*n* = 15)		High BDNF (*n* = 13)	
	*ρ*	*p*	*ρ*	*p*	*ρ*	*p*	*ρ*	*p*
ln (LF)	0.24	0.33	−0.24	0.37	0.88	<0.001^a^	0.34	0.25
ln (HF)	0.12	0.63	−0.19	0.48	0.91	<0.001^a^	0.07	0.83
ln (LF/HF)	0.38	0.12	0.00	0.99	−0.56	0.031	0.07	0.83
ln (total power)	0.18	0.47	−0.16	0.54	0.88	<0.001^a^	0.25	0.42
MHRR	0.54	0.021	−0.10	0.70	0.86	<0.001^a^	0.20	0.51

^a^A significant correlation still existed after controlling for age and sex.

Note: subjects were split into low and high BDNF level, perseveration error, and categories completion groups at the median (8,500 pg/ml, 8.5, and 3.5, respectively).

SERT: serotonin transporter

HRV: heart rate variability

BDNF: brain-derived neurotrophic factor

WCST: Wisconsin card-sorting test

LF: low frequency

HF: high frequency

MHRR: mean heart rate range.

**Table 4:** Spearman’s correlations between SERT availability and HRV indexes in low and high BDNF level groups modulated by the categories completion of WCST.

	SERT availability							
	Low categories completion				High categories completion			
	Low BDNF (*n* = 22)		High BDNF (*n* = 14)		Low BDNF (*n* = 11)		High BDNF (*n* = 15)	
	*ρ*	*p*	*ρ*	*p*	*ρ*	*p*	*ρ*	*p*
ln (LF)	0.63	0.002^a^	0.18	0.53	0.34	0.31	−0.20	0.47
ln (HF)	0.77	<0.001^a^	−0.17	0.56	0.01	0.98	0.02	0.94
ln (LF/HF)	−0.48	0.023	0.41	0.14	0.46	0.15	−0.44	0.10
ln (total power)	0.67	0.001^a^	0.08	0.78	0.31	0.36	−0.13	0.64
MHRR	0.71	<0.001	−0.06	0.84	0.78	0.005	−0.28	0.31

^a^A significant correlation still existed after controlling for age and sex.

Note: subjects were split into low and high BDNF level, perseveration error, and categories completion groups at the median (8,500 pg/ml, 8.5, and 3.5, respectively).

SERT: serotonin transporter

HRV: heart rate variability

BDNF: brain-derived neurotrophic factor

WCST: Wisconsin card-sorting test

LF: low frequency

HF: high frequency

MHRR: mean heart rate range.

## Discussion

Previous studies have demonstrated a correlation between BDNF and serotonin^24^. Our study further showed that a positive correlation existed between serotonin and the ANS in the low BDNF level group. Moreover, the functions of the PFC, represented by the top-down regulatory center during stress and in several neuropsychiatric illnesses^8^, may be modulated by compensatory effects of serotonin and ANS regulation when the PFC function is poor, especially in those with a lower BDNF level.

Substantial evidence has suggested that interaction of BDNF and serotonin may influence neuron structural plasticity^25^, exert genetic interaction effects that alter depression susceptibility^26^, modulate stress-buffering effects^27^, and could even be correlated with risk of depression when facing stressful life events^28^. Also, BDNF and serotonergic neurons have bidirectional effects that modulate their functions^29,30^. Neuro-imaging study has demonstrated a correlation between SERT availability and BDNF level^31^. Our results were in line with previous findings, and showed the existence of a significant interaction between BDNF and SERT.

Furthermore, a positive correlation between SERT and the ANS only existed in subjects with a low BDNF level. Previous studies have demonstrated that effective antidepressant treatment may synergistically enhance the extracellular serotonin level and the BDNF level, therefore improving symptoms of depression and anxiety disorders^32^. Meanwhile, sympathetic-related symptoms can be relieved with enhancement of the extracellular serotonin level^33^. Therefore, in the low BDNF level group, the reduced extracellular serotonin level could be associated with lower ANS function, and vice versa. This result was similar to previous findings in animal models, which showed that 5HT-2A and 5HT-3 receptor activation in the nucleus tractus solitaries regulates ANS function in terms of bradycardia and other vagal activities^34,35^. Interestingly, identical positive correlations between SERT and LF, HF, and total power existed in our study. Although previous studies have suggested that LF might be an indicator of sympathetic and partially parasympathetic regulation, this interpretation is controversial, and recent studies have demonstrated that LF, like HF, also has a predominant parasympathetic function^36^. This indicates that parasympathetic function might play a crucial role in ANS function to modulate symptoms related to an altered serotonin level^37^. The role of parasympathetic activities in our study could be strengthened by MHRR, which is also an index of parasympathetic functioning after deep breathing^38^, and has a positive correlation with SERT. Furthermore, stress-related downregulation of the BDNF level might be partly mediated by the serotonin level^27^ and ANS function^39^ to relieve symptoms. Our study produced similar results, showing that in order to maintain physiological homeostasis, modulations of serotonin and the ANS occur to prevent the detrimental effects of an inadequate BDNF level. Our results may explain why biofeedback management, including muscle relaxation and deep breathing, re-regulates the ANS function and relieves symptoms related to a lower serotonin level (anxiety, palpitations, etc.).

As mentioned, top-down regulation of the PFC to associated neuro-networks could prevent potential damage caused by challenging environments. Therefore, preserved PFC function learned from previous stressful experiences blocks serotonergic activation in the DRN when under stress^40^. On the contrary, one animal study showed that PFC inactivation increased the downstream serotonergic activation in the DRN^41^. Moreover, 5-HT2A receptor activation enhances the working memory function regulated by the dorsolateral PFC^42^. Our findings are consistent with previous findings showing that subjects with poorer PFC function exhibit a significant positive correlation between SERT and ANS function, especially subjects with a low BDNF level. In addition, when subjects have a better PFC function in terms of low perseveration errors or high categories completion on the WCST, the downstream modulating effects of the ANS and serotonin vanish, regardless of BDNF level. Our results implied that preservation of PFC function in order to cope with external stimulation might be sufficient to maintain neuro-physiological homeostasis, but poorer PFC function might switch the control of behavior and emotion to downstream neuro-networks for compensation. When the compensatory effects are disrupted, several psychiatric illnesses could occur, such as major depressive disorder^43^ and schizophrenia^44^, etc. However, interpretation should be performed cautiously, as the compensatory effects may not necessarily be in the form of bottom-up regulation. We do not have any evidence of bottom-up regulation owing to the study design, but the downstream effects for compensation of prefrontal malfunction in our study may be worthy of further exploration in order to prove the bidirectional mechanism in the human brain^45^. Another important result of our study was that in the group of subjects with poorer PFC function, a higher BDNF level seemed to offset the PFC inadequacy. Although the exact mechanisms are unclear, a buffering effect of BDNF in dysregulated cortical function has been reported previously^46^. Therefore, the associations between SERT and the ANS in order to maintain physiological homeostasis in subjects with poorer PFC function may be medicated by a high BDNF level. The exact mechanism merits further investigation.

Several limitations existed in our study, and the results should be interpreted with caution. First, the sample sizes were small, and several factors related to the ANS could not be tested; for example, effects following stratification by gender. Second, although we attempted to avoid several major issues that may influence ANS and SERT functioning, for example, medications use or even BMI^47^, several factors such as lifestyle (including exercise behavior) could not be controlled. Third, the cross-sectional correlation design of our study could not confirm a causal relationship between the ANS and serotonin. Therefore, further challenge tests are required to determine this causal relationship. Fourth, the effects of estrogen might influence the level of serotonin in different menstrual stages^48^. Although we did not control estrogenic effects in our study, previous studies have indicated that the estrogenic effect might have an insignificant influence on SERT availability^49^. Also, individual differences in the equilibrium time and clearance rate of the radiotracer may alter the imaging results. Examination of the metabolism/protein binding ratio of each subject in our study may solve this problem, but limitations with regards to the techniques possible in our laboratory hinder this potential solution. Finally, the model of PFC-serotonin-ANS associations may be over-simplified. A number of studies have shown that other monoamines are also responsible for stress modulation (for example, dopamine and norepinephrine)^1^. Future studies should integrate other monoamines for more complete neuro-network analysis.

**Figure 2:**
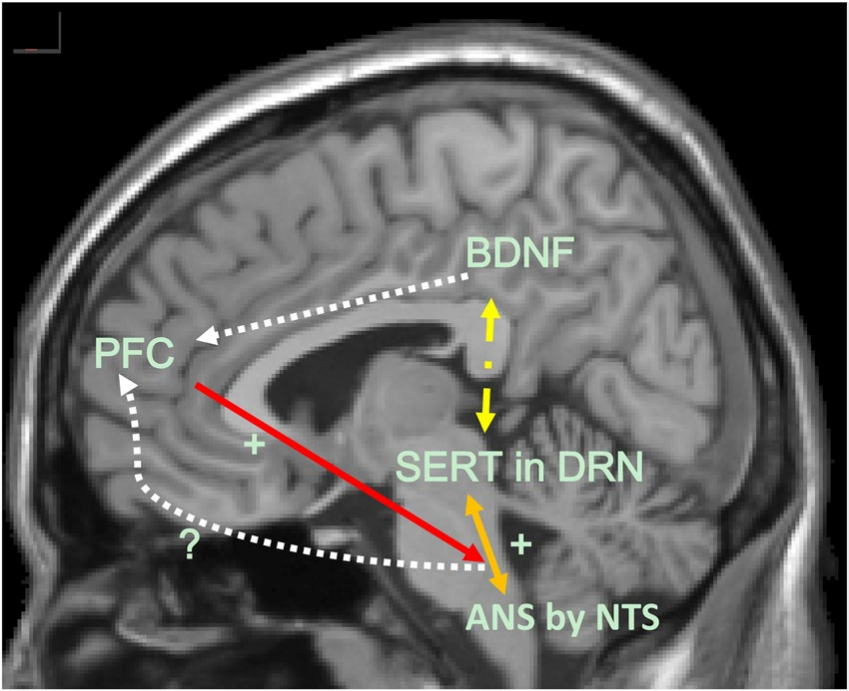
Proposed model of interaction between the PFC, SERT, ANS and BDNF level. Possible top-down regulation from the PFC may modulate downstream associations. PFC: prefrontal cortex. SERT: serotonin transporter. ANS: autonomic nervous system. BDNF: brain-derived neurotrophic factor. DRN: dorsal raphe nucleus. NTS: nucleus tractus solitaries

Our study demonstrated a possible model of cognitive (PFC)-emotional (serotonin)-behavioral (ANS) association in the human brain (see Fig. 2). Malfunction in top-down regulation may be compensated by downstream neuro-network collaborations. Therefore, clinicians should emphasize cognitive rehabilitation in certain neuropsychiatric illnesses for better emotional regulation and improvement of a subject’s ability to cope with stress.

## Methods

### Subjects

Sixty-seven healthy controls, ranging from the age of 18 to 65, evaluated to have no other mental, medical, or neurological problems, were recruited from the community using advertisements for various studies^50,51^. The health of the participants was assessed by a physician, and an experienced psychiatrist used the Chinese version of the Mini International Neuropsychiatric Interview (MINI)^52^ to exclude individuals with any possible psychiatric disorders. Excluded subjects included (i) individuals with medical or mental illnesses identified when examined/interviewed; (ii) pregnant women, or participants suspected to be pregnant, and nursing women; and (iii) patients with drug or alcohol dependence or abuse in the past six months.

No subjects were in receipt of any medication that would affect the study results, including benzodiazepines, antihistamines, beta-adrenergic antagonists, or antidepressants. In addition, participants were not allowed to use alcohol, nicotine, or caffeine on the day of measurement. Before any procedure was performed, written informed consent was obtained from each of the participants after a complete explanation of the study. The Ethical Committee for Human Research at the National Cheng Kung University Hospital approved the study protocol. The methods were carried out in accordance with the relevant guidelines, including any relevant details.

### BDNF measurement

Plasma BDNF level was measured in blood samples taken within a 2-h interval in the morning, between 9 AM and 11 AM, after patients had fasted for at least 8 h. Ten milliliters of whole blood were withdrawn from the antecubital vein of each patient into a vacuum tube containing ethylenediamine tetraacetic acid (EDTA) (Greiner Bio-One Vacuette; Santa Cruz Biotechnology, Santa Cruz, CA, USA), and the blood sample was kept on ice for up to 30 min. To isolate plasma, whole blood was then centrifuged at 3000 × *g* for 15 min at 4 °C and immediately stored at −80 °C. A BDNF kit (Quantikine Human BDNF kit; R&D Systems, Minneapolis, MN, USA) and an enzyme-linked immunosorbent assay (ELISA) reader (Spectra-Max-M2; Molecular Devices, Sunnyvale, CA, USA) were used to analyze the level of plasma BDNF, the minimum detectable dose of which is typically < 80 pg/ml for humans. All samples were assayed in duplicate by laboratory staff blind to patient status.

### SERT availability in the midbrain

For the SPECT examination with [^123^I]ADAM, the protocol, intravenous administration time of the radioligand, MRI co-registration, and reasons for selecting the midbrain as the region of interest (ROI) have been described in our previous studies^50,53^.

## HRV measurements

### ANS activity

A full 20-minute period of recumbent acclimatization in a quiet room that was maintained at a comfortable temperature (25–27 °C) preceded the cardiovascular measurements, which started at 10 AM. Subjects were asked to relax and breathe normally to avoid hyperventilation. Cardiac autonomic function was calculated by the geometric method, which is based on short-term measurements of the interbeat interval (IBI)^54^. Briefly, the sequence of IBI (IBI_1_, IBI_2_, …, IBI_n_) was transformed into a figure on a two-dimensional plane by plotting IBI_k+1_ against IBI_k_. The length of the transverse axis (T) is affected by both the sympathetic and parasympathetic blockades, whereas the length of the longitudinal axis (L) is affected only by the parasympathetic blockade. Thus, log10 (L × T) is a cardiac vagal index, whereas the L/T ratio is a cardiac sympathetic index. These two indices have been demonstrated to be more reliable than conventional measures including spectral analysis^54^. This geometric method has been found to be a sound measurement^55^, and its advantages include: (i) controlled respiration or other maneuvers are not required, and (ii) as few as 100 IBIs are sufficient for the assessment^54^.

Power spectral density analysis of HRV was performed by fast Fourier transformation^56^. Several spectral components were defined as follows: low frequency (LF) (0.04 to 0.15 Hz), HF (0.15 to 0.40 Hz), and total power (≤0.4 Hz). The very low frequency (VLF) data were excluded, because the available VLF data in short-term recording is dubious and should be avoided^57^. The total power represents the total autonomic activity. The HF power of HRV represents an index of cardiac parasympathetic (vagal) activity, whereas the LF power represents an index of vasomotor sympathetic activity, or both sympathetic and vagal activities. The LF/HF ratio has been proposed as an index of the relative balance of sympatho-vagal influences on the heart, with higher LH/HF ratios reflecting increased sympathetic activity or decreased parasympathetic modulation^58^. These indexes were ln-transformed to correct the skewness.

### Autonomic function during resting

The beat-to-beat blood pressure (BP) of the left radial artery and the heart rate were monitored for 5 minutes while subjects remained in the supine position. BP and heart rate were continuously monitored using a Tonometry BP Monitor (Colin BP-508, Colin Co., Komaki-City, Aichi, Japan) and input into a computer console. The referential BP was recorded using a sphygmomanometer cuff over the right brachial artery and measured at intervals of 2.5 minutes. Whenever the tonometry BP measurement was questionable or failed, cuff measurement for calibration was automatically started.

### Autonomic function during the respiratory challenge test

The subjects were asked to take a deep breath and the heart rate was continuously recorded. Each deep breath cycle contained a five-second inspiration and a five-second expiration; 5 successive breath cycles were measured in one assessment. We subtracted the minimum HR during expiration from the maximum HR during inspiration for each cycle of breathing, the time interval between two cycles being one minute, and then determined the mean of the differences. The differences were recorded as the mean heart rate range (MHRR), which is one of the most widely-used methods by which to assess HRV under respiratory challenge testing^59^.

### WCST measurements

During the WCST, patients were required to match response cards to four stimulus cards, along with one of three dimensions (color, form, or number) on the basis of verbal feedback (correct or wrong) without being given any information about the dimensions. Once the subject had sorted a series of 10 cards by one category, they were then asked to sort the cards again by a different category. There were 64 cards in the present test. All definitions of indices are described in the WCST manual^60^. There are many measures by which to determine WCST performance. In this study, we selected the number of WCST categories that patients completed and the perseveration errors as indexes of WCST performance.

### Statistical analysis

First, multiple regression was used to test the interactions of BDNF level and SERT availability with each individual HRV index, within which the HRV indexes were the dependent variables, and SERT, BDNF, and interaction were independent variables. Subjects were further split into high and low BDNF groups by the median for further stratified correlation analysis. Spearman’s rho correlation and partial correlation, controlling for age and sex, were both used to examine the correlations between SERT availability and the HRV indexes in different groups. Independent t tests and Chi-square tests were used to assess differences between groups.

Because the results of the WCST (categories completed and perseveration errors) might moderate the aforementioned results, median-splitting of categories completed or perseveration errors was conducted. Correlations between SERT availability and HRV indexes in subjects with differing WCST performances and BDNF levels were probed. The data were analyzed using Statistical Package for Social Science software version 17 (SPSS Inc., Chicago, IL, USA). The threshold for statistical significance was set at *p* < 0.05.

## Electronic supplementary material

Supplementary Table S1. Full results of interactions of BDNF level and SERT availability with ANS function.
