# Supplementary material for: Prefrontal cortex modulates the correlations between brain-derived neurotrophic factor level, serotonin, and the autonomic nervous system
Source: Sci Rep. 2018 Feb 7;8:2558. doi: 10.1038/s41598-018-20923-y (PMC5803248; doi:10.1038/s41598-018-20923-y)
Supplement: Supplementary file 1 — Supplementary Table S1. Full results of interactions of BDNF level and SERT availability with ANS function. [file 41598_2018_20923_MOESM1_ESM.doc]

Prefrontal cortex modulates the correlations between brain-derived neurotrophic factor level, serotonin, and the autonomic nervous system

Wei Hung Chang, I Hui Lee, Mei Hung Chi, Shih-Hsien Lin, Kao Chin Chen, Po See Chen, Nan Tsing Chiu, Wei Jen Yao, Yen Kuang Yang

Supplementary Table S1. Full results of interactions of BDNF level and SERT availability with ANS function.

| Outcome | Predictors | Total  (*n* = 67) | | Low perseveration error  (*n* = 34) | | High perseveration error  (*n* = 28) | | Low categories completion  (*n* = 36) | | High categories completion  (*n* = 26) | |
| --- | --- | --- | --- | --- | --- | --- | --- | --- | --- | --- | --- |
|  |  | *F*^a^ | *p* | *F*^a^ | *p* | *F*^a^ | *p* | *F*^a^ | *p* | *F*^a^ | *p* |
| ln (LF) | BDNF | 3.07 | 0.08 | 2.13 | 0.15 | 3.22 | 0.09 | 4.01 | 0.054 | 1.19 | 0.29 |
|  | SERT | 8.63 | 0.005 | 2.67 | 0.11 | 11.34 | 0.003 | 10.99 | 0.002 | 1.20 | 0.29 |
|  | BDNF*SERT | 4.00 | 0.0497 | 2.14 | 0.15 | 4.57 | 0.043 | 4.87 | 0.035 | 1.11 | 0.30 |
| ln (HF) | BDNF | 4.16 | 0.046 | 1.71 | 0.20 | 6.32 | 0.019 | 13.24 | 0.001 | 0.23 | 0.64 |
|  | SERT | 7.06 | 0.010 | 1.37 | 0.25 | 12.63 | 0.002 | 16.82 | 0.000 | 0.02 | 0.90 |
|  | BDNF*SERT | 4.20 | 0.045 | 1.69 | 0.20 | 6.06 | 0.021 | 12.48 | 0.001 | 0.23 | 0.64 |
| ln (LF/HF) | BDNF | 1.18 | 0.28 | 0.00 | 0.96 | 2.55 | 0.12 | 9.26 | 0.005 | 7.98 | 0.010 |
|  | SERT | 0.40 | 0.53 | 0.22 | 0.64 | 2.03 | 0.17 | 4.49 | 0.042 | 4.52 | 0.045 |
|  | BDNF*SERT | 0.65 | 0.42 | 0.00 | 0.97 | 1.40 | 0.25 | 6.91 | 0.013 | 7.60 | 0.012 |
| ln (total power) | BDNF | 4.09 | 0.047 | 2.06 | 0.16 | 5.05 | 0.034 | 6.31 | 0.017 | 1.18 | 0.29 |
|  | SERT | 8.89 | 0.004 | 2.02 | 0.17 | 14.85 | 0.001 | 14.80 | 0.001 | 0.76 | 0.39 |
|  | BDNF*SERT | 4.53 | 0.037 | 1.75 | 0.20 | 6.44 | 0.018 | 6.92 | 0.013 | 0.88 | 0.36 |
| MHRR | BDNF | 6.97 | 0.010 | 4.43 | 0.044 | 4.49 | 0.045 | 7.24 | 0.011 | 5.20 | 0.033 |
|  | SERT | 10.30 | 0.002 | 6.10 | 0.019 | 9.21 | 0.006 | 10.38 | 0.003 | 5.28 | 0.032 |
|  | BDNF*SERT | 6.42 | 0.014 | 3.81 | 0.06 | 5.35 | 0.030 | 7.07 | 0.012 | 4.88 | 0.038 |

BDNF: brain-derived neurotrophic factor; SERT: serotonin transporter; ANS: autonomic nervous system; LF: low frequency; HF: high frequency; MHRR: mean heart rate range.
